# Adherence of community caretakers of children to pre-packaged antimalarial medicines (HOMAPAK^®^) among internally displaced people in Gulu district, Uganda

**DOI:** 10.1186/1475-2875-5-40

**Published:** 2006-05-15

**Authors:** Jan H Kolaczinski, Naptalis Ojok, John Opwonya, Sylvia Meek, Andrew Collins

**Affiliations:** 1 Malaria Consortium, Africa Regional Office, Sturrock Road, Kampala, Uganda; 2 Disease Control and Vector Biology Unit, London School of Hygiene & Tropical Medicine, Keppel Street, London, UK; 3 District Health Services, Gulu District, Uganda; 4 Malaria Consortium, Head Office, Leonard Street, London, UK

## Abstract

**Background:**

In 2002, home-based management of fever (HBMF) was introduced in Uganda, to improve access to prompt, effective antimalarial treatment of all fevers in children under 5 years. Implementation is through community drug distributors (CDDs) who distribute pre-packaged chloroquine plus sulfadoxine-pyrimethamine (HOMAPAK^®^) free of charge to caretakers of febrile children. Adherence of caretakers to this regimen has not been studied.

**Methods:**

A questionnaire-based survey combined with inspection of blister packaging was conducted to investigate caretakers' adherence to HOMAPAK^®^. The population surveyed consisted of internally displaced people (IDPs) from eight camps.

**Results:**

A total of 241 caretakers were interviewed. 95.0% (CI: 93.3% – 98.4%) of their children had received the correct dose for their age and 52.3% of caretakers had retained the blister pack. Assuming correct self-reporting, the overall adherence was 96.3% (CI: 93.9% – 98.7%). The nine caretakers who had not adhered had done so because the child had improved, had vomited, did not like the taste of the tablets, or because they forgot to administer the treatment. For 85.5% of cases treatment had been sought within 24 hours. Blister packaging was considered useful by virtually all respondents, mainly because it kept the drugs clean and dry. Information provided on, and inside, the package was of limited use, because most respondents were illiterate. However, CDDs had often told caretakers how to administer the treatment. For 39.4% of respondents consultation with the CDD was their reported first action when their child has fever and 52.7% stated that they consult her/him if the child does not get better.

**Conclusion:**

In IDP camps, the HBMF strategy forms an important component of medical care for young children. In case of febrile illness, most caretakers obtain prompt and adequate antimalarial treatment, and adhere to it. A large proportion of malaria episodes are thus likely to be treated before complications can arise. Implementation in the IDP camps now needs to focus on improving monitoring, supervision and general support to CDDs, as well as on targeting them and caretakers with educational messages. The national treatment policy for uncomplicated malaria has recently been changed to artemether-lumefantrine. Discussions on a suitable replacement combination for HBMF are well advanced, and have raised new questions about adherence.

## Background

Prompt and effective treatment of malaria remains a challenge for malaria control programmes. From the moment a patient experiences the first symptoms of malaria to the point when the disease has been cured, many potential barriers have to be overcome. These include delays in recognition of symptoms and treatment seeking, and non-adherence to the drug regimen. Helping caretakers of young children to overcome these and other barriers to prompt and effective treatment is of particular importance in sub-Saharan Africa. In this malaria endemic region, severe malaria is the most common cause of death of young children. The greatest proportion of these deaths is due to the lack of early recognition of malaria symptoms (which overlap with those of other fatal febrile illnesses in early childhood) and their management with prompt and effective treatment within 24 hours of onset of symptoms [1-5].

In most of the highly malaria endemic areas of sub-Saharan Africa, malaria is initially treated at home with drugs purchased from the private sector [6-9]. Uganda was no exception to this prior to 2002 [10-12], which is when the Ministry of Health (MoH) introduced the Home Based Management of Fever (HBMF) strategy [13]. HBMF is based on the Home Based Management of Malaria strategy developed by Roll Back Malaria [14,15]. The key components are: i) effective communication to enable caretakers to recognise malaria illness early and to take appropriate action, ii) ensuring that Community Drug Distributors (CDDs) and health service providers have the skills to manage malaria illness, iii) ensuring availability and access to effective and preferably pre-packaged antimalarial drugs, and iv) providing a mechanism for supervision and monitoring. The potential for HBMF to reduce under-five mortality and progression to severe malaria is enormous [16,17].

Three years after its introduction in Uganda HBMF covers almost the whole country, though it is at various stages of implementation. The current treatment of choice is a pre-packaged combination of sulfadoxine-pyrimethamine (SP) and chloroquine (CQ) with the trade name HOMAPAK^®^, though it has recently been decided to change it. Discussions on a suitable replacement combination are well advanced. Distribution relies on volunteer CDDs that are trained and provided with a supply of HOMAPAK^®^. Monitoring and supervision of this activity is the responsibility of the nearest health facility.

Evaluations have been carried out in a number of districts [18,19], but none have attempted to examine adherence to the drug regimen. Building on previous support of the Malaria Consortium to the HBMF implementation in the northern districts of Gulu, Kitgum and Pader [20], the present work was carried out to study the adherence to HOMAPAK^® ^in caretakers of children among internally displaced persons (IDP) in this region. The results are meant to contribute to the evidence-base of HBMF in Uganda, allowing for adjustment of the strategy where necessary.

## Methods

### Study area

The study was conducted in Gulu district in the northwest of Uganda. The district comprises five sub-districts, Aswa, Kilak, Omoro, Gulu and Nwoya. The centre is the town of Gulu. The region is inhabited by the Acholi tribe, which speaks a local language called Luo.

Gulu and the neighbouring districts of Kitgum and Pader have suffered from 18 years of conflict caused by the activities of a rebel group, the Lord's Resistance Army (LRA). Activities are directed against the central government, but often target the civilian population [21]. As a result, more than two million people have been displaced and are now living in camps for internally displaced persons (IDPs). Most camps were established next to the bases of the Uganda's People Defence Force (i.e. the national army) and houses are grouped very closely together, to provide better protection from LRA attacks. This has resulted in over-crowded living conditions.

Recent data for Gulu district shows that there are presently 460,000 IDPs living in 53 recognised camps [22]. The older, better-established camps have a health unit and trained community owned resource persons (CORPs). Inhabitants of more recently established camps are generally required to travel to the nearest camp with a health unit for medical assistance or to consult a CORP trained in health, where available. A number of different cadres of CORPs have been trained in health related activities. Those trained in HBMF are referred to as community drug distributors (CDDs). As is the case for all CORPs, the work of CDDs is meant to be voluntary, but facilitated though incentives provided by the community and the MoH [19].

Malaria is endemic over most of Uganda, with varying levels of endemicity. In the IDP population of Gulu district, malaria/fever is the main cause of death in children under 5 years, comprising an estimated 25.3% of self-reported mortality [21]. Aggregated annual data on outpatient attendance collected through the health management information system (HMIS) also indicates that malaria poses a considerable burden on this district. Treatment for malaria constitutes about 45% of all outpatient attendance; in children below the age of five years this figure reaches approximately 60% (Office of the District Director of Health Services, Gulu; unpublished data).

### Antimalarial medication and distribution

**Figure 1:**
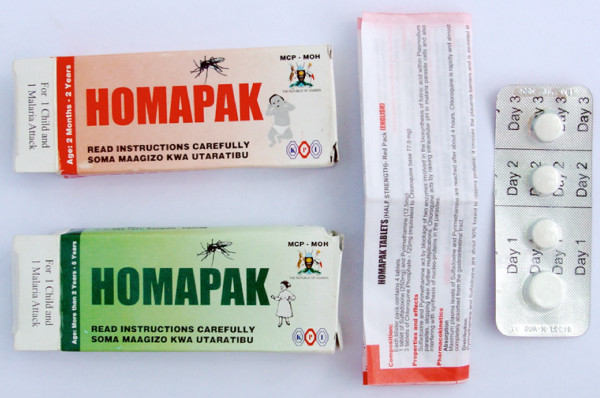
Examples of HOMAPAK^® ^blister packs for children age 2 – 23 months (red package) and 24 – 59 months (green package).

The HBMF strategy relies on the use of a pre-packaged anti-malarial treatment, called HOMAPAK^®^. Each treatment course consists of one tablet of sulfadoxine-pyrimethamine (SP) and three tablets of chloroquine (CQ), delivered in a blister pack and to be taken over three consecutive days (SP + CQ on day 1). For ease of use, the blister pack has been labelled 'Day 1, Day 2, Day 3' and has a perforation between the doses for each of the days (figure 1). To provide the right dose for weight, HOMAPAK^® ^has been made available in two different packs with different doses per tablet, one for children age two months to 23 months (red package) and one for children age 24 to 59 months (green package). The outside of the packages and the blister packs are labelled with a dosing schedule in English, and a leaflet inside the package provides detailed instructions in English, Kiswahili, Ateso, Luganda, Luo and Runyakitara. It is manufactured by Kampala Pharmaceutical Industries (1996) Ltd., P.O. Box 7551, Kampala, Uganda.

HOMAPAK^® ^is distributed free of charge at community level through the public health system. In the IDP camps, the stock is kept at the health units and issued to CDDs when required. To monitor distribution, CDDs have been issued with a patient register book. The records are meant to be compiled on a monthly basis at the nearest health units and to be forwarded to the office of the district director of health services (DDHS).

### Inclusion criteria

All caretakers listed in the CDDs' registers as having received HOMAPAK^® ^for their child, more than 3 days but less than one week ago, were included in the study. The one-week window was meant to ensure that the treatment had only recently been completed and that the respondent was still able to recall the details of seeking treatment for this episode of her/his child's illness. It was also meant to increase the chances that the blister pack had not been disposed off and could be inspected by the surveyor, to verify whether all of the tablets had been taken.

### Sampling

Selection of camps was based on accessibility within one day and on security considerations. LRA activity in the district is still considerable and makes certain camps inaccessible, whereas travel to others is only possible with an armed escort. Eight camps were selected and visited on separate days (Table 1). At the time of the survey, all the selected camps were accessible without an armed escort.

**Table 1:** List of camps surveyed and their characteristics

**Name of Camp**	**Sub-District**	**Distance from Gulu Town (km)**	**Population**	**Households**	**HOMAPAK Drug Distributors**	**Health Units**
Unyama	Aswa	11	11,221	3,064	20	√
Teyapadhola	Aswa	32	5,395	1,445	11	√
Paicho	Aswa	24	7,968	2,142	17	√
Awer	Kilak	20	14,293	3,247	22	√
Lacor	Kilak	14	5,371	1,345	2	×
Pabbo	Kilak	40	40,870	9,396	58	√
Palenga	Omoro	16	8,859	1,971	13	√
Pagak	Kilak	24	6,694	2,150	18	×

At least one day before the survey of a camp, the district radio station broadcasted a message to inform the camp leader, health staff and the CDDs of the survey, to request their presence at the health units at 10 a.m. and to ask any caretakers that had obtained HOMAPAK^® ^during the last week to stay at home during the morning. Most camp inhabitants regularly listen to the radio and this method is commonly used to convey messages in this remote area.

On arrival in each camp, the four surveyors and the study investigator were introduced and the purpose of the study was outlined. Together with each CDD, his/her registers were screened for last month's activities. As the register has no column to record the date, surveyors together with the CDDs worked their way backwards through the register to establish which patients met the inclusion criteria. The CDD and one surveyor then visited each of these households. If the caretaker or a suitable respondent was present and if these agreed to participate in the study an interview was conducted. If the HOMAPAK^® ^blister pack had been retained by the household it was inspected for remaining tablets. If no suitable respondent was present in a household the next one in the register was visited. After visiting all potential study participants from one register, the surveyor returned to the health units and repeated the process with another CDD. This process was continued until all the registers of the CDDs that had come to meet the survey team had been screened.

### Data collection

A four page (26 questions) semi-structured questionnaire was developed in English. It was designed to cover aspects of adherence, treatment seeking, packaging and malaria related knowledge. For consistency, the Luo words for specific terms describing time of day or symptoms of illness were agreed upon by the surveyors and included in the questionnaire. Surveyors were trained on the use of the questionnaire and requested to ask questions in an open way. All surveyors spoke fluent English and Luo. Questions were asked in Luo and responses recorded under the appropriate sections of the questionnaire.

Prior to each interview, the surveyors introduced themselves and the study, based on a pre-formulated introduction, and asked caretakers for their consent. To determine whether a CDD had provided the correct dose of HOMAPAK^®^, his/her record of the age of the child and the colour of the pack distributed (red or green) were compared. For children with a recorded age of 2 years, both red and green HOMAPAK^® ^were considered as correct, as it was impossible to determine the exact age of the child. Caretakers rarely have birth certificates for their children and it must thus be assumed that they give an approximate age when they report to the CDD.

Apart from administering the questionnaire and inspecting the blister pack, no other methods for measuring drug usage and adherence (e.g. blood and urine assays) were employed, due to logistic and funding limitations. A number of studies have shown that carer-reported drug histories can be reliable [23-25], though results of other investigators did not confirm this [26]. For verification, inspection of blister packs was chosen, where possible.

### Definition of adherence

Children whose caretakers showed a blister pack that still contained drugs at the time of the home visit were considered non-adherent, if the questionnaire confirmed that treatment had been received more than 3 days ago and should, therefore, have been completed. In cases where the caretaker presented an empty blister pack and stated that all medication had been used to treat the same child, or where no blister pack was presented and the caretaker stated that the course had been completed, the patient was considered compliant.

### Data analysis

Questionnaires were checked for completeness and consistency at the end of each field day. For some questions an open option had been included to record the respondent's answer if it did not fit into the pre-determined categories. These answers were screened and coded into as many additional categories as seemed appropriate. The data were then entered into the study database in Microsoft Excel. Once the study had been completed, the data file was checked for completeness before exporting it to STATA 9.0 (Stata Corp., USA 2005). Further checks on the completeness of the data were performed in STATA 9.0 prior to analysis. Sample characteristics and patient's classification were described as proportions and, where deemed appropriate, presented with 95% confidence intervals (CI). Variations between the camps were not investigated, as this had not been part of the data analysis plan. Numbers of observations were also too small for some of the camps to allow meaningful stratified analysis.

## Results

### Summary of study population

From 12 to 19 September 2005, 243 caretakers were approached in eight IDP camps, none of whom refused to take part in the study. Of these, 226 (93%) were mothers, six (2.5%) were fathers, five (2.1%) were grandmothers and the remainder were other relatives. Two of the mothers were found to be still treating their children, because they were visited less than three days after obtaining treatment. They were excluded from further analysis. The mean age of children that had been treated with HOMAPAK^® ^was 2.5 years (95% CI: 2.4 – 2.7).

### HOMAPAK^® ^dosage and adherence

95.9% of children (CI: 93.3% – 98.4%) received the correct dose of HOMAPAK^® ^for their age (Table 2). Though we did not systematically investigate reasons for providing the wrong dose, discussion with CDDs provided evidence that, at least in some cases, they had run out of the correct packages.

**Table 2:** Proportion of children that received the correct treatment dose and adherence of their caretakers to the treatment regimen

**Name of Camp**	**Correct Package of HOMAPAK^® ^Received (%)**	**Blister Pack Retained (%)**	Overall Adherence of Caretakers (%)*
Unyama	27 (100)	19 (70.4)	24 (88.9)
Teyapadhola	19 (90.5)	17 (81.0)	20 (95.3)
Paicho	37 (94.9)	22 (56.4)	36 (92.3)
Awer	18 (100)	8 (44.4)	17 (94.4)
Lacor	8 (80.0)	5 (50.0)	10 (100)
Pabbo	59 (96.7)	16 (26.2)	61(100)
Palenga	19 (90.5)	11 (52.4)	20 (95.2)
Pagak	44 (100)	28 (63.6)	44 (100)

**Total**	231 (95.9)	126 (52.3)	232 (96.3)

* Combined data from caretakers that had retained the blister pack and those that had not, but reported to have used all the drugs to treat the same child

Out of all the caretakers interviewed, 126 (52.3%) had retained the blister pack. Adherence in this group was 92.9%, whereas adherence reported by caretakers that had not retained the blister pack was 100%. Overall adherence was thus 96.3% (CI: 93.9% – 98.7%), assuming correct reporting by caretakers (Table 2). Of the nine caretakers who had not completed the course for their child, four stated that they had stopped because the child got better, three forgot to administer the treatment, one had stopped because the child had vomited and one because the child did not like the taste of the tablets.

### Treatment seeking behaviour and effects of treatment

Caretaker's reports of seeking treatment within 24 hours did not always match the records of the CDDs. In 208 (86.3%) cases, the CDD's records on treatment seeking were consistent with the statement provided by the caretakers, who overall reported that 85.5% would seek treatment within 24 hours (Table 3).

**Table 3:** Treatment seeking behaviour and effects of treatment

**Name of Camp**	Treatment Seeking Within 24 Hours (%)*	Recovered After Treatment (%)**	No Adverse Reaction to Treatment (%)**
Unyama	21 (77.8)	23 (95.8)	23 (95.8)
Teyapadhola	21 (100)	16 (100)	16 (100)
Paicho	32 (82.1)	27 (87.1)	22 (78.6)
Awer	17 (94.4)	14 (100)	16 (100)
Lacor	9 (90.0)	10 (100)	10 (100)
Pabbo	50 (82.0)	34 (85.0)	33 (100)
Palenga	17 (81.0)	17 (89.5)	17 (100)
Pagak	39 (89)	36 (97.3)	36 (100)

**Total**	206 (85.5)	177 (92.7)	173 (96.1)

* As stated by the caretaker (data from CDD register not shown)

** Proportions in this column are based on available records for this category.

Of the 35 (14.5%) caretakers who delayed seeking treatment for their child, it was most common to state that they either had medicines at home (25.7%) or that they had thought the illness would resolve itself (25.7%). The third most common reply was that the CDD had not been available at the time (20.0%). Other responses were that they had no time (8.6%), had bought other medicine first (8.6%) or were away from home when the child got sick (5.7%).

As part of their reporting, CDDs should register the outcome of HOMAPAK^® ^treatment and whether any adverse reactions occurred. To complete these two columns, follow-up of the patient is required. Inspections of the registers showed that this had often not been done. For 50 (20.8%) and 61 (25.3%) of patients the respective columns for outcome or adverse reaction had not been completed. For the cases where records were available, these showed that 14 (5.8%) patients had been referred by the CDD and that 177 (92.7%) children had recovered (Table 3).

Adverse reactions seemed the most problematic to record, as CDDs were often unclear of the definition or how to record it (the options are "yes/no", but seem to have been confused in some cases where all patients had been recorded as having suffered from adverse reactions). The data in Table 3 were compiled after seeking some clarification from the CDDs, but still have to be interpreted with caution, particularly as the majority of adverse reactions were reported from one camp (Paicho). However, overall it seems as thought the CQ + SP combination causes few side effects in young children.

### Use of printed treatment information and perception of blister pack

Use of the dosage chart and other treatment information printed on the pack and on the leaflet inside the pack was limited, largely because most caretakers (87.8%) could not read. 36 (14.9%) respondents stated that they had read the information on the pack and only 20 (8.3%) that they had read the inserted leaflet (Table 4). Apart from being unable to read, people did not consult the leaflet because of the small writing, lack of time or because the text was perceived as being too complicated. Of those that stated that they had read the information on the pack, all found it useful. Similarly, 90% of the few respondents that had read the inserted leaflet reported that they found it useful.

**Table 4:** Caretakers use of printed information

**Name of Camp**	**Read Information On Pack (%)**	**Read Information Inside Pack (%)**
Unyama	6 (22.2)	4 (14.8)
Teyapadhola	6 (28.6)	4 (19.1)
Paicho	4 (10.3)	1 (2.6)
Awer	3 (16.7)	2 (11.1)
Lacor	3 (30.0)	0
Pabbo	7 (11.5)	3 (4.9)
Palenga	1 (4.8)	1 (4.8)
Pagak	6 (13.6)	65 (11.4)

**Total**	36 (14.9)	20 (8.3)

Though only a small proportion of caretakers made use of the information material provided with HOMAPAK^®^, it was found that 47.0% of the respondents that had not read any of the information had been told by the CDD how to administer the drug. An additional three respondents stated that they already knew how to use HOMAPAK^®^. Whether all other caretakers used the medication without having obtained instructions cannot be judged from the present data. Observation during interviews seemed to suggest that caretakers were familiar with its use. To clarify what role the CDDs play in educating caretakers and whether caretakers obtain treatment information through other channels (e.g. neighbours), future investigations should include a question on this aspect.

When asked whether the blister packing itself was perceived as being useful, all respondents apart from one who was undecided replied that they approved of the pack. The majority (92.5%) mentioned that they like the blister packaging because it keeps the drugs clean and/or dry. Other frequently quoted reasons were that it makes the drugs easier to use (32.9%) and provides an exact dosage (28.8%). A few respondents stated that it helps them to report to the CDD or the health units what type of medication their child has received in the past (1.3%), that it kept the drugs safe (2.5%) or that they like it because it has instructions on it (2.1%).

### Knowledge of signs and symptoms of malaria, and response to signs of fever

Investigation into the awareness of malaria symptoms showed that 95.4% of caretakers associated fever with malaria. However, many other less specific symptoms, such as diarrhoea, vomiting or feeling dizzy, were also frequently mentioned. As a first response to fever, caretakers said they mainly use what they referred to as 'tepid sponging' (58.1%) or go to see the CDD (39.4%). In discussion it emerged that respondents actually wash their child with cold water rather than use 'tepid sponging'.

Generally, action is taken either immediately (56.0%) or after a few hours (37.3%). When the condition of the child does not improve, many caretakers said that they again consult with the CDD (52.7%) to get further advice or be referred to hospital. Others stated that they would go directly to the health units (36.9%) or to the hospital (10.4%).

## Discussion

The present study shows that CDDs play an important role in the control of malaria in IDP camps, some of them providing presumptive malaria treatment for febrile illness to more than 60 children per month. Many are highly motivated despite limited or no support or incentives (most CDDs were not regularly supervised and had received only one bar of soap in the last two years). Some even took the initiative to improve on the HOMAPAK^® ^register by adding columns to record the date and name of the caretaker. Despite the overall positive impression of the current implementation of HBMF, the visit of the survey team was often seen as an occasion for the CDDs to request more support. Promises that had been made during their training have clearly not been met. This observation is consistent with that of other studies [19], who highlighted the need for further technical support and for delivery of promised incentives.

Fortunately, even in the absence of basic support, HBMF was working well. Most caretakers sought advice within 24 hours of their children falling ill with fever, were provided with the correct treatment and adhered to it. The bitter taste of the chloroquine tablets did not seem to affect adherence. Only one of the mothers reported that the treatment was not completed because her child had not liked the taste. In any case, sugar-coating of tablets would only hide the bitter taste when tablets are administered whole, but some mothers said that they crush the tables before giving it to their babies or young children. By far the most important reasons for not adhering were that caretakers had observed that their child had recovered or that they had forgotten to give the drug (which may have been due to the fact that the child had got better). This shows that it is necessary to encourage caretakers to complete the treatment course, which may be achieved by making them and CDDs more aware of the risks of non-adherence (i.e. therapeutic failure and selection for drug resistance).

As far as could be judged from the registers and observations, most patients had recovered and few had experienced any adverse effect. Unfortunately, recording of data on these latter aspects was often incomplete, because patients had not been followed-up and/or because CDDs were unclear as to the exact definition of an adverse reaction. The observed lack of attention to patient follow-up and reporting could be resolved through regular supervision, feedback and refresher training. Prior to the planned introduction of a new combination therapy, it would therefore be useful to revise the exiting register, to include columns for the date and for the name of the head of household. Introduction of the amended register could then be used to provide refresher training on reporting, health education, etc. and to clearly define adverse reactions.

Though the observed and reported adherence of caretakers to HOMAPAK^® ^was reassuring, there is room for improvement on knowledge and practices related to malaria. Many caretakers clearly associated fever with malaria, but also did so with a large number of other signs and symptoms. In the absence of fever, these may not indicate infection with *Plasmodium *spp., but may be the result of another, equally dangerous, childhood disease. For HBMF, it is important to continue health education on the importance of fever, its diagnosis and the need to seek prompt treatment. To avoid confusion of caretakers (and CDDs) it will be important to ensure that these messages are kept simple and are consistent with those delivered through other programmes.

Further education will also be required on the use of tepid sponging. As a first response to febrile illness many wash their child with cold water, which may worsen rather than improve the fever. Educational materials should be developed to outline simple methods of tepid sponging, particularly on how to raise the water temperature on a cool night (e.g. by putting a damp cloth on the caretaker's body before sponging the child). Education should be targeted at caretakers and CDDs, as the latter are an important source of information particularly for illiterate people. An option may be to provide CDDs with illustrated flip-charts to help them to better relay information to the caretakers.

None of the caretakers stated that they used traditional healers, either as an immediate source of treatment when their child has fever or if the child does not improve. However, discussions with the surveyors at the end of each survey day showed that they had noticed that some of the children had small incisions on their chest or other signs that traditional healers had been consulted during the child's life. The present survey tool and method were not suitable to investigate the role of traditional healers in the IDP camps. More research on this subject is encouraged, to establish whether traditional healers are consulted in the treatment of febrile illness in the IDP camps, whether they may contribute to delays in consulting CDDs and if they can be brought into the HBMF strategy. Previous work in western Uganda indicates that local illness categorisation may exclude some fevers from being treated with 'western' treatment [27]. This may result in delayed and under-treatment of potential malaria.

The results on the acceptability of the blister-pack are consistent with results from a study in western Uganda prior to the introduction of HOMAPAK^® ^[28]. The authors found that 90.5% of women preferred pre-packed treatment over loosely packed tablets. The most mentioned reasons for this preference were safety and cleanliness. As in the present study, it was observed that few respondents mentioned correct dosing as an important aspect of pre-packaged drugs. With reference to results from a study by the same first author [29], which showed that more than 81% of rural mothers knew the correct dose of CQ treatment for an adult, and 37% knew it for a 3 year old child, it was suggested that a high proportion of caretakers are confident about dosing but find cleanliness more important. Whether caretakers in the IDP population are aware of correct CQ + SP dosages for their children was not investigated, but the conclusion that pre-packaged drugs are clearly acceptable [28] certainly also applies to the IDP population. The main reason for this seems to be cleanliness and it may be worthwhile to highlight other positive aspects in future education campaigns. The use of pre-packaging to guide dosing will certainly become more important and useful when new drug regimens (e.g. ACTs) with more tablets and more frequent doses, are introduced.

Methodological considerations of importance in interpreting results from the present study are its generalizability and potential biases. For the following reasons the results may not apply to other rural areas of Uganda that are unaffected by conflict. In IDP camps people live closer together than in rural villages, which reduces travel to reach a CDD. In areas not affected by the chronic emergency, caretakers may take longer to seek treatment, CDDs may spend more time away due to better security and employment opportunities, and follow-up of patients may be even lower than in IDP camps. Fewer caretakers may also seek treatment from CDDs because they have more alternatives, such as private practitioners, drug shops or traditional healers.

The camps sampled were all relatively close to the town of Gulu, in areas that have comparatively good security. In camps that are located in areas that were not accessible to the survey team or that could not have been reached and surveyed within one day, the implementation of the HBMF strategy may be weaker. However, even within the easily reachable camps we observed that drug supply and implementation was not always as good as might be expected, mainly because the persons in charge of the health units did not feel sufficiently responsible to facilitate the work of the CDDs and to ensure a constant HOMAPAK^® ^supply. Based on our limited observations, the success of HBMF is largely dependent on the motivation of staff in charge of the local health units and on the CDDs, but not on distance from the district centre.

The survey relied heavily on the cooperation of CDDs to identify and find caretakers. At least 60% of the CDDs came to meet the survey team at the health units and were extremely motivated and cooperative. However, some failed to come because they had to attend to other duties and others just did not show-up. The latter may not have heard the announcement on the radio. As it was also noticed, some individuals that came to meet the survey team were less motivated than others. They were generally not seeing many patients, had limited interaction with the ones they saw and were not completing their registers with care. Also, despite a request for caretakers to remain home during the morning, many had left to attend to their fields and were not available for an interview. All of the above factors may have introduced bias, potentially towards better adherence than may have been observed if camps had been selected at random and been revisited until all caretakers had been interviewed.

## Conclusion

In IDP camps the HBMF strategy forms an important component of medical care for young children. In case of febrile illness, most caretakers obtain prompt and adequate antimalarial treatment, and adhere to it. It is expected that a large proportion of malaria episodes are thus treated before complications can arise. Implementation of the strategy now needs to focus on improving monitoring, supervision and general support to CDDs and by targeting them and caretakers with educational messages. There is a need to realize that heavy reliance on community volunteers does not make HBMF self-sustaining and that its future success depends on ongoing commitment from the MoH and international donors.

Discussions on a suitable replacement combination for HBMF are well advanced, and have raised new questions about adherence. Further operational research will be required to establish how more complicated treatment regimens can best be delivered by CDDs.

## Authors' contributions

JHK and AC conceived of the study, participated in its design and coordination, and drafted the manuscript. JHK supervised all field activities and analysed the data. NO and JO participated in the design of the study and its coordination at field level. SM provided technical input into the study design and data analysis, and contributed to drafting the manuscript. All authors read and approved the final manuscript.
